# Dietary supplements do not improve bone morphology or mechanical properties in young female C57BL/6 mice

**DOI:** 10.1038/s41598-022-14068-2

**Published:** 2022-06-13

**Authors:** Amy Creecy, Collier Smith, Joseph M. Wallace

**Affiliations:** 1 Department of Biomedical Engineering, Indiana University-Purdue University Indianapolis, SL 220B, 723 W. Michigan St., Indianapolis, IN 46202 USA; 2 Department of Anatomy, Cell Biology, and Physiology, Indiana University School of Medicine, Indianapolis, IN USA

**Keywords:** Nutrition, Bone

## Abstract

Bone is a hierarchical material formed by an organic extracellular matrix and mineral where each component and their physical relationship with each other contribute to fracture resistance. Bone quality can be affected by nutrition, and dietary supplements that are marketed to improve overall health may improve the fracture resistance of bone. To test this, 11 week old female C57BL/6 mice were fed either collagen, chondroitin sulfate, glucosamine sulfate, or fish oil 5 times a week for 8 weeks. Femurs, tibiae, and vertebrae were scanned with micro-computed tomography and then mechanically tested. Glucosamine and fish oil lowered elastic modulus, but did not alter the overall strength of the femur. There were no differences in bone mechanics of the tibiae or vertebrae. Overall, the data suggest that dietary supplements did little to improve bone quality in young, healthy mice. These supplements may be more effective in diseased or aged mice.

## Introduction

Bone is a hierarchical material with the nanoscale level consisting of an organic extracellular matrix (ECM) scaffolding on which the mineral hydroxyapatite forms^1^. Collagen accounts for 90% of the organic ECM, and the remaining matrix proteins including osteocalcin, osteopontin, glycosaminoglycans, and proteoglycans such as biglycan^2^, are simply referred to as non-collagenous proteins Collagen primarily consists of the amino acids hydroxyproline, proline, and glycine and undergoes post-translational modifications, such as when it is crosslinked by the enzyme lysyl oxidase^3^. In addition to protein and mineral, water is present and can be either free within the many pores in bone, or more closely affiliated with collagen or mineral in what is referred to as bound water ^4^. Alterations in one component of the matrix often alter the other phases given their intimate interaction. As an example, mutations in collagen affecting its structure can lead to alterations in mineralization, and non-collagenous proteins may affect the amount of bound water in bone^5^.

Each nanoscale component contributes to the overall fracture resistance of bone, which is determined by both structural mechanical properties that are dependent on bone mass, and tissue or material-level properties that are independent of structure. Tissue and material-level properties are often collectively referred to as measures of bone quality. High levels of mineralization increase the stiffness of the matrix and the overall strength, but can also lead to a decrease in the bone’s ability to dissipate energy^6,7^. The organic portion of the ECM is thought to be primarily responsible for the post-yield properties, with destruction of the organic ECM components resulting in brittle bones^8^. While a certain level of crosslinking is needed to maintain bone strength, an overabundance of crosslinking can increase bone’s brittleness. Enzymatic crosslinking is a highly controlled process, but the accumulation of AGEs, which can increase with age, diabetes, and under pro-inflammatory conditions^9–11^, can be detrimental to mechanical integrity. Non-collagenous proteins such as glycosaminoglycans assist with fracture prevention by increasing the levels of bound water in bone which may facilitate better load sharing between collagen and mineral^5^. Alterations to the bone matrix, including losses in bound water or mineral, or an increase in collagen crosslinking, can drive increased fracture risk.

Two strategies for improving bone’s fracture resistance throughout an individual’s lifetime are to increase bone mass and quality as much as possible during adolescence and to prevent bone loss with age. Adequate nutrition is one way to promote bone formation during development and to prevent bone loss with age^12^. Nutritional requirements include the components of the bone matrix such as calcium, phosphorus, magnesium, and protein, but there are other important factors to consider, such as the essential vitamins that serve as cofactors for enzymes involved with organic ECM formation^13^.

In recent years, over the counter dietary supplements have become plentiful, and many are marketed as being able to improve cartilage, bone, and general health. Chondroitin sulfate is a major subtype of glycosaminoglycan found in bone and glucosamine sulfate is a hexosamine sugar that is required to form many macromolecules, including glycosaminoglycans^14^. Both supplements have been studied for relief of osteoarthritis-associated pain^15,16^. There is some in vitro evidence to suggest they may act through inhibition of nuclear factor kappa B (NF-κB) signaling^17,18^. NF-κB promotes osteoclastogenesis and increases inflammatory markers^19^. Chondroitin sulfate and glucosamine can be absorbed by the gut and have been observed to be increased after supplementation in the serum^20,21^. However, the bioavailability is variable and can be low as there is loss that occurs in the gut^22,23^. Collagen is the main protein component of bone, and hydrolyzed collagen supplements are marketed for improvement of skin quality. Hydrolyzed collagen has previously been shown clinically to prevent loss of BMD in post-menopausal women^24^ but did not alter bone turnover markers in adult men given the supplement after exercise^25^. Hydrolyzed collagen would be absorbed from the gut as simple amino acids such as glycine, hydroxyproline, and proline. These amino acids can be readily absorbed in the gut through hydrolyzed collagen supplements^26^. Fish oil is recommended for a variety of outcomes, including improving cardiovascular health and cancer prevention. Fish oil contains omega-3 poly-unsaturated fatty acids which may provide an anti-inflammatory effect^27^, and there is some evidence it may decrease bone resorption^28^. Fish oil can be absorbed from the gut as n-3 poly-unsaturated fatty acids^29^.

Given the difficulty of obtaining measures of bone structure and mechanical properties in clinical studies, preclinical models are needed to fully determine the effect of dietary supplements on bone quantity and tissue quality. The preclinical evidence to date has varied as to whether or not supplements can improve bone’s fracture resistance. There are some indications that feeding rodents fish oil may improve bone quality^30^, but studies in rabbits indicated a possible detrimental effect of fish oil on bone^31^. Rats fed hydrolyzed collagen showed improvements in trabecular bone^32^. Intradermal injections with chondroitin sulfate improved bound water and bone mechanics in mice^5^. More detailed investigations of the various supplements marketed to improve bone health are needed. To this effect, we tested whether treatment with the dietary supplements collagen, chondroitin sulfate, glucosamine sulfate, or fish oil would improve bone’s structural parameters and tissue-level mechanical properties in growing, female mice.

## Methods

### Animals

Female C57BL/6 mice bred in house were used for this study (male and female C57BL/6NHsd breeders purchased from Envigo (Indianapolis, IN)). Mice were housed 5 per cage, fed water and standard chow (LabDiet 5001, 28.7% kcal protein, 13.4% kcal fat and 57.9% kcal carbohydrates) ad libitum, and were kept on 12 h light cycle. Females were used in this study as women have a higher incidence of fracture than men^33^. Starting at 11 weeks of age, animals (*n* = 10/group) were randomly assigned to groups and given dietary supplements of either hydrolyzed type I collagen (Nature’s Truth Ultra Collagen) at 1 g/kg, glucosamine sulfate potassium chloride (Spectrum, G1296) at 300 mg/kg, chondroitin sulfate sodium salt (Spectrum, C1610) at 250 mg/kg, or fish oil (Spectrum, F1192) at 1 g/kg. These values were calculated using a body surface area (BSA) calculation^34^ from human dosage values of 81.1 mg/kg for collagen, 24.3 mg/kg for glucosamine, 20.3 mg/kg for chondroitin sulfate and 81.1 mg/kg for fish oil. A mass of 21.5 g, calculated as the midpoint between the expected average weights for mice at the beginning and end of the study, was used to calculate dosage for all mice. Mice were treated starting at 11 weeks to determine if supplements that may be incorporated into the bone matrix would have an effect during growth. Bacon-flavored nutritional supplements Bacon Softies™ (Bio-Serv, F3580-1, 23.4% kcal protein, 28.7% kcal fat, 47.8% kcal carbohydrates) were used as a vehicle for delivering the dietary supplements. Individual pieces were cut to the same size. Dietary supplements were suspended in MilliQ grade water, except for fish oil. The suspended dietary supplements were adsorbed onto the surface of the bacon treat. MilliQ grade water was used for control mice. The Bacon Softies were placed within cages, and mice were observed to ensure each animal was eating the treats. Animals were given dietary supplements five times a week for a total of eight weeks. Body weights were monitored weekly. Animals were euthanized at 19 weeks of age by CO_2_ inhalation. Cervical dislocation was performed afterwards as a secondary method to confirm euthanasia. All animal protocols were approved by the Indiana University Purdue University Indianapolis Institutional Animal Care and Use Committee (IACUC). All procedures and methods were performed in accordance with ARRIVE guidelines and IACUC guidelines. Femurs, tibiae, and vertebrae were harvested, stripped of soft tissue, wrapped in phosphate buffer saline (PBS) soaked gauze, and stored at − 20 °C until further analysis.

### Micro-computed tomography

#### Femurs

Right femurs were scanned on a Skyscan 1172 micro-CT system (60 kV, 167 µA, 0.5 mm Al filter) with a voxel size of 9.8 µm, a rotation step of 0.7° and frame averaging of 2. A hydroxyapatite standard was scanned once a week for calculation of tissue mineralization density. After reconstruction, images were rotated so that the mid-shaft of the bone was vertically-straight and the orientation of the bones were consistent with regards to the direction of the anterior and medial surfaces. Cortical bone at the mid-shaft was analyzed centered at the point 75% of the distance between the bottom of the trochanter and the top of the distal growth plate, starting 75% of the distance away from the growth plate. Trabecular bone is not present at this location. Using a custom Matlab code, seven slices were used to measure cortical bone parameters including total cross-sectional area, marrow area, cortical area, bone area fraction, cortical thickness, periosteal circumference, endosteal circumference, minimum moment of inertia (Imin), maximum moment of inertia (Imax), moment of inertia about the medial lateral axis and cortical tissue mineral density (Ct.TMD). Trabecular bone in the metaphysis was analyzed starting at the most proximal end of the distal growth plate and extending proximally by 1 mm. An auto-contouring program using vendor-supplied software (CTan) was used to isolate trabecular bone from the surrounding cortical bone. Trabecular bone properties including bone volume fraction (BV/TV), trabecular thickness (Tb.Th), trabecular number (Tb.N), trabecular spacing (Tb.Sp), and trabecular tissue mineralization density (Tb.TMD) were calculated using CTan. The surrounding cortical bone was not analyzed in this region. Length measurements were measured from left femurs using a caliper.

#### Tibiae

Right tibiae were scanned using the same parameters as femurs (9.8 µm voxel size) and were rotated such that the tibiae was aligned vertically-straight. A 1 mm portion of the metaphysis was analyzed starting immediately below the end of the proximal growth plate and extending distally. Seven slices of cortical bone were analyzed at 50% of the length of the tibiae. Cortical and trabecular bone parameters were calculated as described for femurs above. Right tibiae were measured for length prior to scanning with calipers.

#### Vertebrae

L4 vertebrae were removed from the spinal column. The intervertebral discs (IVD) on the cranial and caudal surfaces were removed. Vertebrae were scanned with the same parameters as femurs, and analyzed for the same trabecular properties. Reconstructed scans were rotated so that the cranial side of the vertebrae was facing up and the vertebrae posterior surface was vertically straight. The trabecular bone of the vertebrae was analyzed from where the vertebrae was fully formed on the cranial surface to the top of the growth plate on the caudal side. Contours were drawn by hand in this region.

### Mechanical testing

#### Three-point bending tests for femurs

Femurs were tested to failure in three-point bending with the anterior side in tension using a span of 7.6 mm. The displacement rate was 0.025 mm/sec and bones were hydrated with deionized (DI) water during testing. Load and displacement were recorded. To take into account the geometry of the bone and to determine estimated material properties, these were mapped to stress and strain using standard engineering beam theory Equations^35^ and CT data (moment of inertia about the medial–lateral axis and extreme fiber distance to the anterior surface). The yield point was determined using the 0.2% offset method based on the slope of the stress/strain curve. Data were analyzed for structural mechanical properties from the load–displacement curve including yield force, ultimate force, yield displacement, post-yield displacement, total displacement, stiffness, yield work, post-yield work, and total work. Additionally, estimated material properties including yield stress, ultimate stress, yield strain, total strain, elastic modulus, resilience, and toughness were calculated from the stress–strain curve.

#### Fracture toughness testing for tibiae

Right tibiae were hand-notched on the anteromedial aspect of the tibia at approximately 50% of the bone length using a scalpel coated with 0.5 µm diamond suspension solution. The notch did not exceed the depth of the cross-sectional midpoint. These bone were tested in three-point bending with the notched side in tension at a displacement rate of 0.001 mm/sec while hydrated with DI water. The notch was centered in between the bottom span supports. Load and displacement were measured. Upon breaking, the marrow was flushed and bones were dehydrated in a graded series of ethanol. Bones were stored under a vacuum overnight at room temperature, sputter-coated with gold, and imaged using a scanning electron microscope to capture notch geometry of the fracture surface. The notch edge was used to calculate the angle of crack initiation and the instability edges were used to calculate the angle at which unstable crack growth occurred. This calculation was performed in Matlab with the user choosing the points of the notch edge and the point where unstable crack growth occurred on the SEM image of the notch. The angle of the notch was then calculated based on the location of the bone’s cross-sectional centroid. This method of calculations can help correct for differences in notch geometry that occur with user notching. Critical stress factors based on the behavior of thin walled cylinders were calculated for the point of crack initiation (Kcinitial), where the max force occurred (Kcmax), and when the instability growth occurred (Kcinstability) as described in depth elsewhere^36^.

#### Compression testing for vertebrae

L4 vertebrae were loaded in compression at a rate of 0.025 mm/sec to a displacement distance of 3.5 mm using custom-designed testing fixtures. The bottom portion of the fixture contained a post to pass through the neural canal between the body of the vertebra and vertebral processes, holding the bone in place. The top loading point had a small flat ellipsoid surface to apply load directly to the vertebral body. Because of potential asymmetry in the flatness of the end plate, the initial load may not have been equally distributed across the vertebral body and could have resulted in slight toe-in behavior. Stiffness was determined as the slope in the first linear portion of the curve after this toe-in (if present). Peak force was determined as the highest force reached during the test, and was normalized to the bone volume fraction calculated from the trabecular contour. Given the lack of a breaking failure point for trabecular bone and the difficulty in defining yield, only peak force, stiffness, and normalized peak force were reported.

### Statistical analysis

D’Agostino & Pearson tests were used to determine normality. If the data set was normal, one-way ANOVA was used to analyze the data, otherwise the Kruskal–Wallis test was used. An alpha of 0.05 was set. In cases of significance from the ANOVA, multiple comparisons were made using Dunnett’s multiple comparisons test to compare treated animals to the control group. In cases of significance from the Kruskal–Wallis test, multiple comparisons were made using Dunn’s multiple comparisons test to compare treated animals to the control group. Pearson correlations coefficients between cortical TMD and modulus of the femur were made using GraphPad.

## Results

### Global parameters

There were no differences in final body weight measured at the end of the study, indicating that any differences in structural properties of the bone would not be the result of differences in body weight (Table 1).

**Table 1:** Global and femur structural properties. Asterisks indicate significant differences when compared to control group.

Property	Control (*n* = 10)	Collagen (*n* = 10)	Chondroitin sulfate (*n* = 10)	Fish oil (*n* = 10)	Glucosamine (*n* = 10)	*p* value
**Global**						
Body weight (g)	23.6 ± 1.0	23.9 ± 1.8	23.0 ± 1.5	24.2 ± 1.7	24.2 ± 1.2	0.3077
**Cortical bone**						
Length (mm)	14.7 ± 0.2	14.8 ± 0.3	14.7 ± 0.3	14.9 ± 0.2	14.8 ± 0.4	0.4534
Total cross-sectional area (mm^2^)	1.64 ± 0.09	1.65 ± 0.15	1.60 ± 0.09	1.72 ± 0.06	1.74 ± 0.12	0.0224
Marrow area (mm^2^)	0.812 ± 0.053	0.828 ± 0.096	0.790 ± 0.055	0.862 ± 0.048	0.890 ± 0.076*	0.0161
Cortical bone area (mm^2^)	0.826 ± 0.040	0.823 ± 0.066	0.806 ± 0.046	0.855 ± 0.033	0.852 ± 0.042	0.1097
Bone area fraction (%)	0.505 ± 0.012	0.499 ± 0.018	0.505 ± 0.016	0.498 ± 0.016	0.490 ± 0.011	0.1486
Cortical thickness (mm)	0.215 ± 0.007	0.213 ± 0.009	0.212 ± 0.009	0.217 ± 0.007	0.214 ± 0.003	0.6903
Periosteal circumference (mm)	5.27 ± 0.14	5.28 ± 0.24	5.20 ± 0.13	5.39 ± 0.10	5.42 ± 0.18	0.0263
Endosteal circumference (mm)	3.91 ± 0.11	3.95 ± 0.21	3.89 ± 0.12	4.03 ± 0.11	4.10 ± 0.15*	0.0135
Imax (mm^4^)	0.216 ± 0.027	0.218 ± 0.040	0.206 ± 0.022	0.236 ± 0.018	0.239 ± 0.029	0.0479
Imin (mm^4^)	0.120 ± 0.010	0.122 ± 0.020	0.114 ± 0.013	0.131 ± 0.010	0.134 ± 0.017	0.0324
Ct.TMD (g/cm^3^ HA)	1.34 ± 0.02	1.33 ± 0.03	1.35 ± 0.02	1.35 ± 0.02	1.35 ± 0.02	0.6218
**Trabecular bone**						
BV/TV (%)	7.15 ± 0.77	8.35 ± 2.03	7.74 ± 1.93	7.11 ± 1.35	6.68 ± 1.28	0.1498
Tb.N (1/mm)	1.16 ± 0.10	1.40 ± 0.33	1.32 ± 0.33	1.12 ± 0.15	1.12 ± 0.21	0.0358
Tb.Sp (mm)	0.285 ± 0.018	0.276 ± 0.029	0.278 ± 0.028	0.297 ± 0.023	0.294 ± 0.026	0.6712
Tb.Th (mm)	0.0619 ± 0.0048	0.0598 ± 0.0023	0.0585 ± 0.0032	0.0632 ± 0.0054	0.0599 ± 0.0052	0.1289
Tb.TMD (g/cm^3^ HA)	0.693 ± 0.029	0.684 ± 0.014	0.678 ± 0.021	0.702 ± 0.034	0.685 ± 0.025	0.2899

All values are reported as average ± standard deviation. Overall ordinary one way ANOVA or Kruskal Wallis p values are reported.

### Femurs

Several cortical bone parameters in the right femur differed with treatments as determined by overall ANOVA or Kruskal–Wallis tests including cross-sectional area, marrow area, periosteal circumference, endosteal circumference, Imax, and Imin. Only glucosamine-treated mice had differences when compared individually with controls, displaying a larger endosteal circumference (*p* = 0.0202) and marrow area (*p* = 0.0461). In the distal metaphysis, Tb.N was significantly different by one-way ANOVA (*p* = 0.0358), but individual comparisons indicated no differences.

**Figure 1:**
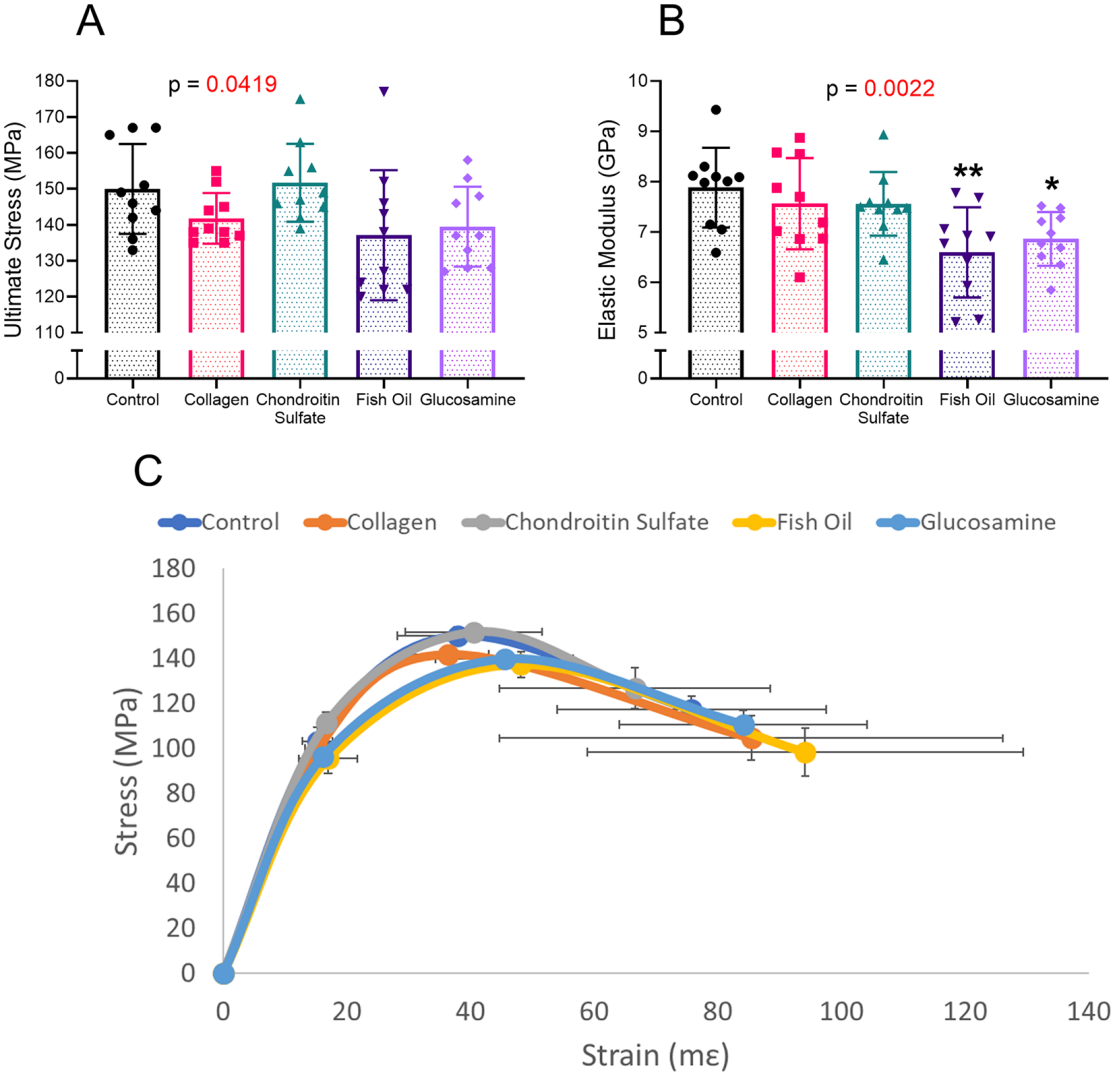
Mechanics of the femur. Ultimate stress (**A**), elastic modulus (**B**) and schematics of stress–strain curves (**C**). Overall ordinary one way ANOVA or Kruskal Wallis p values are listed above the graph. Asterisks indicate significance when the individual group below was compared to the control group in a posthoc analysis with * = *p* = 0.0169, ** = *p* = 0.0019.

Most mechanical properties were unchanged with any treatments (Fig. 1, Table 2). There was a difference in ultimate stress, but no posthoc differences between treatment groups and the controls emerged (Fig. 1A). There was also a difference in elastic modulus, with fish oil and glucosamine having a reduced elastic modulus versus controls (Fig. 1B). Modulus did not correlate with TMD, as observed in Supplemental Fig. 1. There were no other differences in the mechanical properties of the femurs, as seen in Table 2.

**Table 2:** Mechanics of femurs.

Property	Control (*n* = 10)	Collagen (*n* = 10)	Chondroitin sulfate (*n* = 10)	Fish oil (*n* = 10)	Glucosamine (*n* = 10)	*p* value
**Structural**						
Yield force (N)	11.0 ± 2.1	10.8 ± 1.5	11.5 ± 1.1	11.0 ± 2.2	11.1 ± 1.8	0.9300
Ultimate force (N)	16.0 ± 1.4	15.6 ± 2.1	15.8 ± 1.4	15.9 ± 2.2	16.1 ± 1.1	0.9618
Yield displacement (µm)	119 ± 20	121 ± 16	133 ± 11	132 ± 36	123 ± 17	0.5210
Post-yield displacement (µm)	473 ± 172	555 ± 324	394 ± 166	601 ± 278	518 ± 144	0.3199
Total displacement (µm)	592 ± 166	676 ± 323	526 ± 161	733 ± 276	641 ± 139	0.3131
Stiffness (N/mm)	107 ± 10	103 ± 8	98 ± 4	98 ± 13	103 ± 6	0.1085
Yield work (mJ)	0.753 ± 0.243	0.750 ± 0.169	0.855 ± 0.132	0.850 ± 0.344	0.791 ± 0.209	0.7463
Post-yield work (mJ)	6.49 ± 2.10	7.09 ± 3.92	5.42 ± 2.21	7.49 ± 2.58	7.21 ± 1.87	0.4293
Total work (mJ)	7.24 ± 2.07	7.84 ± 3.90	6.27 ± 2.17	8.34 ± 2.57	8.00 ± 1.83	0.4357
**Material**						
Yield stress (MPa)	103 ± 20	100 ± 20	111 ± 16	95 ± 21	96 ± 17	0.4925
Yield strain (mε)	15.2 ± 2.5	15.2 ± 2.0	16.6 ± 1.2	17.0 ± 4.7	16.1 ± 2.3	0.5969
Total strain (mε)	75.8 ± 21.8	85.4 ± 40.7	66.6 ± 21.9	94.2 ± 35.2	84.1 ± 20.0	0.2897
Resilience (MPa)	0.90 ± 0.29	0.88 ± 0.24	1.04 ± 0.18	0.95 ± 0.40	0.90 ± 0.25	0.7256
Toughness (MPa)	8.63 ± 2.30	8.83 ± 4.08	7.55 ± 2.56	9.23 ± 2.73	9.12 ± 2.31	0.7144

Asterisks indicate significant differences when compared to control group. All values are reported as average ± standard deviation. Overall ordinary one way ANOVA or Kruskal Wallis p values are reported.

### Tibiae

**Figure 2:**
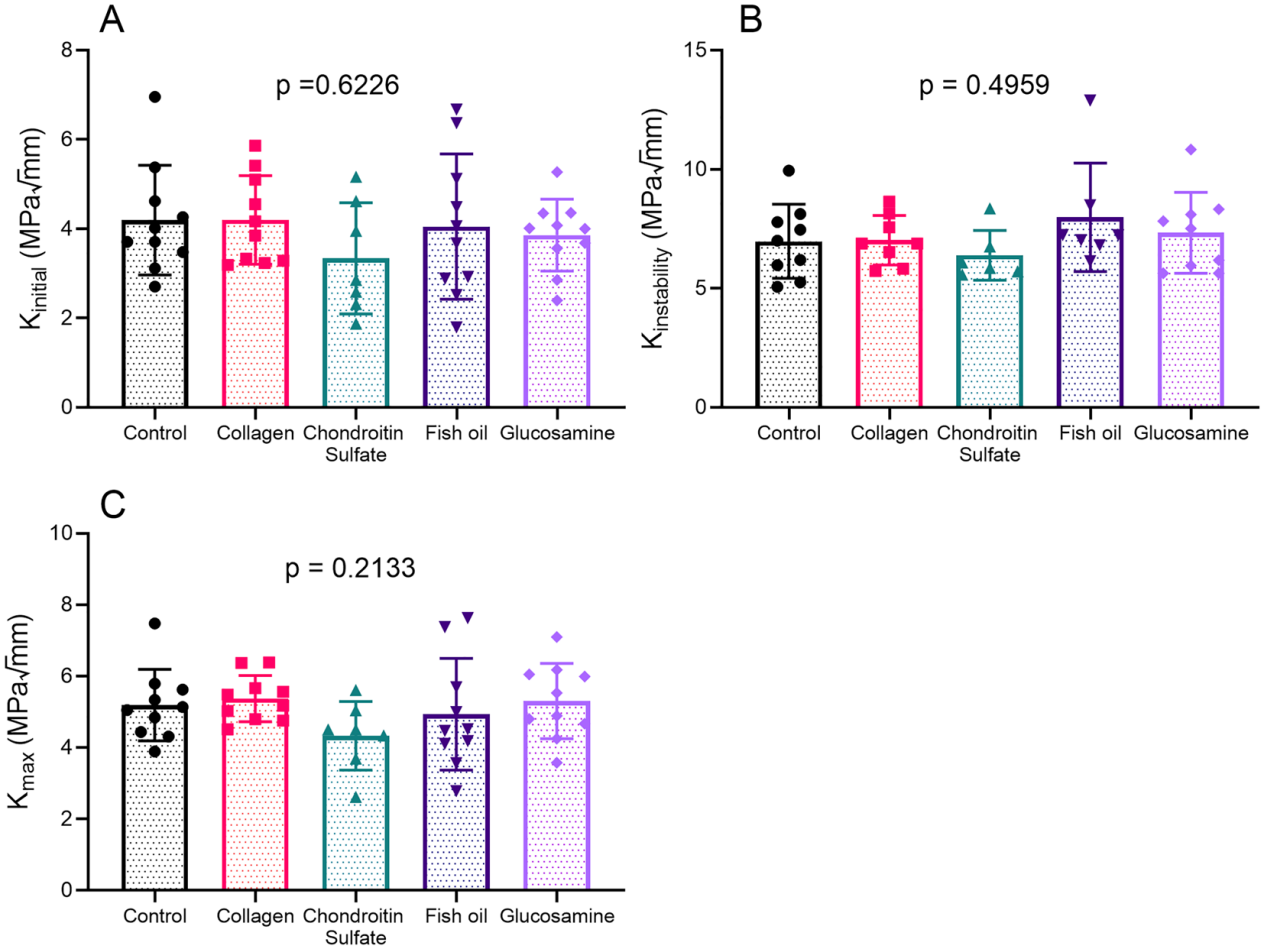
Fracture toughness parameters of tibiae of critical stress factors (**A**) initial energy, (**B**) instability energy, and (**C**) max energy. Overall ordinary one way ANOVA or Kruskal Wallis p values are given above graphs.

Few properties differed in the tibiae of treated mice. Chondroitin sulfate treated mice had lower trabecular thickness in the tibiae metaphysis than controls (Table 3). There were no differences in cortical bone morphology (Table 3), nor were there differences in any of the 3 fracture toughness measures (Fig. 2).

**Table 3:** Structural properties of the tibiae.

Property	Control (*n* = 9–10)	Collagen (*n* = 10)	Chondroitin sulfate (*n* = 10)	Fish oil (*n* = 10)	Glucosamine (*n* = 10)	*p* value
**Cortical bone**						
Length (mm)	17.8 ± 0.2	17.9 ± 0.3	17.3 ± 2.0	18.1 ± 0.4	18.2 ± 0.6	0.3716
Total cross-sectional area (mm^2^)	1.02 ± 0.05	1.02 ± 0.10	0.96 ± 0.07	1.06 ± 0.07	1.04 ± 0.07	0.0707
Marrow area (mm^2^)	0.367 ± 0.032	0.365 ± 0.051	0.338 ± 0.043	0.391 ± 0.047	0.377 ± 0.025	0.0694
Cortical area (mm^2^)	0.651 ± 0.030	0.655 ± 0.053	0.626 ± 0.035	0.667 ± 0.035	0.661 ± 0.044	0.2130
Bone area fraction (%)	0.640 ± 0.017	0.643 ± 0.021	0.650 ± 0.027	0.631 ± 0.024	0.637 ± 0.009	0.3174
Cortical thickness (mm)	0.227 ± 0.007	0.228 ± 0.009	0.226 ± 0.010	0.227 ± 0.008	0.228 ± 0.009	0.9749
Periosteal circumference (mm)	4.33 ± 0.12	4.31 ± 0.21	4.21 ± 0.18	4.39 ± 0.17	4.37 ± 0.14	0.1226
Endosteal circumference (mm)	2.72 ± 0.13	2.71 ± 0.18	2.63 ± 0.16	2.82 ± 0.15	2.77 ± 0.10	0.0663
Imax (mm^4^)	0.0821 ± 0.0088	0.0812 ± 0.0160	0.0752 ± 0.0094	0.0888 ± 0.0109	0.0881 ± 0.0115	0.0746
Imin (mm^4^)	0.0668 ± 0.0072	0.0686 ± 0.0124	0.0593 ± 0.0091	0.0709 ± 0.0109	0.0671 ± 0.0094	0.1261
Ct.TMD (g/cm^3^ HA)	1.35 ± 0.01	1.36 ± 0.02	1.37 ± 0.03	1.36 ± 0.02	1.35 ± 0.02	0.5074
**Trabecular bone**						
BV/TV (%)	9.81 ± 0.77	11.47 ± 2.40	10.37 ± 2.59	9.59 ± 0.65	9.72 ± 2.64	0.2299
Tb.N (1/mm)	1.47 ± 0.13	1.77 ± 0.44	1.64 ± 0.40	1.46 ± 0.08	1.51 ± 0.44	0.2070
Tb.Sp (mm)	0.303 ± 0.032	0.281 ± 0.046	0.297 ± 0.040	0.307 ± 0.022	0.300 ± 0.050	0.6326
Tb.Th (mm)	0.0668 ± 0.0031	0.0654 ± 0.0030	0.0631 ± 0.0027*	0.0659 ± 0.0029	0.0645 ± 0.0020	0.0491
Tb.TMD (g/cm^3^ HA)	0.994 ± 0.047	0.986 ± 0.043	0.977 ± 0.038	0.979 ± 0.040	0.970 ± 0.037	0.7424

Asterisks indicate significant differences when compared to control group. All values are reported as average ± standard deviation. Overall ordinary one way ANOVA or Kruskal Wallis p values are reported.

### Vertebrae

**Figure 3:**
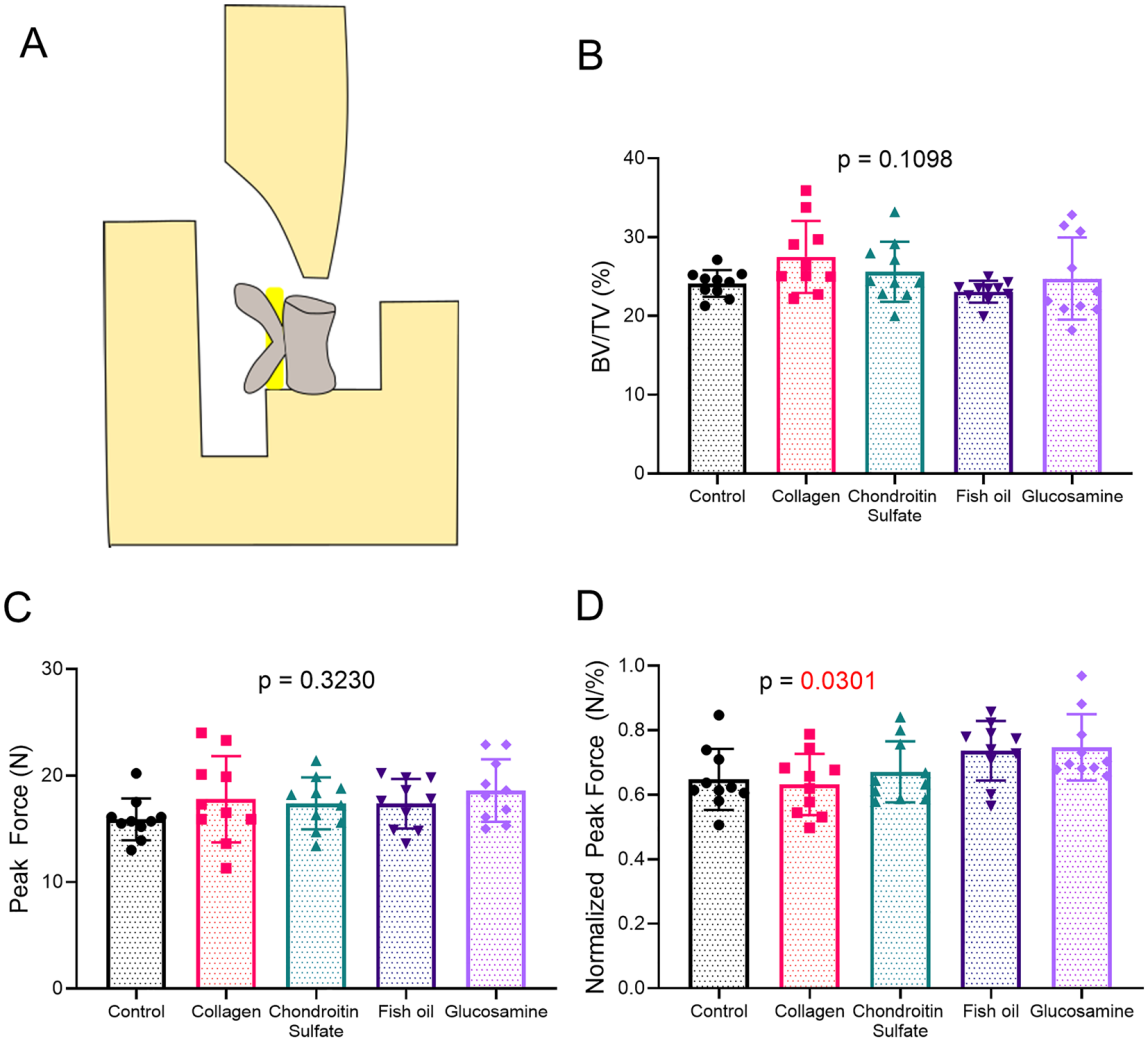
Compression testing of vertebrae. Schematic of compression fixtures with vertebrae (**A**), bone volume fraction of vertebral trabecular bone (**B**), peak force during compression (**C**), and peak force normalized to the bone volume fraction of vertebral trabecular bone (**D**). Overall ordinary one way ANOVA or Kruskal Wallis p values are given above graphs.

There were no differences in trabecular architecture or mechanical properties in the vertebrae of treated mice when compared to controls (Fig. 3, Table 4).

**Table 4:** Structural and mechanical properties of the vertebrae.

Property	Control (*n* = 10)	Collagen (*n* = 10)	Chondroitin sulfate (*n* = 10)	Fish oil (*n* = 10)	Glucosamine (*n* = 10)	*p* value
Tb.N (1/mm)	3.70 ± 0.27	4.13 ± 0.61	3.93 ± 0.53	3.52 ± 0.20	3.83 ± 0.74	0.0553
Tb.Sp (mm)	0.221 ± 0.012	0.205 ± 0.024	0.211 ± 0.026	0.229 ± 0.015	0.217 ± 0.029	0.1732
Tb.Th (mm)	0.0653 ± 0.0015	0.0665 ± 0.0023	0.0651 ± 0.0019	0.0657 ± 0.0014	0.0644 ± 0.0023	0.1814
Tb.TMD (g/cm^3^ HA)	0.778 ± 0.009	0.782 ± 0.018	0.780 ± 0.013	0.781 ± 0.007	0.767 ± 0.015	0.0876
Stiffness (N/mm)	30.7 ± 8.2	31.4 ± 13.4	33.3 ± 12.7	35.4 ± 13.7	30.5 ± 14.4	0.9569

Asterisks indicate a significant difference when compared to control. All values are reported as average ± standard deviation. Overall ordinary one way ANOVA or Kruskal Wallis p values are reported.

## Discussion

Our findings indicate that dietary supplements had little impact on bone morphology or mechanics in young female mice and cannot be used to improve bone’s fracture resistance. Bone quality, inferred from material-level mechanical properties and fracture toughness, did not improve with treatment. The only alteration in bone quality was a decrease in elastic modulus with glucosamine or fish oil, which is considered negative and would not be advantageous in preventing fracture. However, while the modulus was lower, there were no differences in ultimate stress for either of these groups. This suggests that the bones were less resistant to applied loading, but this did not affect the overall strength of the tissue. Modulus was altered in these bones without a change in tissue mineralization, which is an important factor in modulus as shown when mechanical testing have been done on different types of bones with varying mineralization^7,37^. This suggests that the mineral composition itself may have been altered with the treatments. Fracture toughness parameters, measurements of the ability of bone to resist crack initiation and growth, also did not change with treatment. Together, these data suggest that adding more of the basic components of the bone matrix in the diet of growing mice does not improve the quality of the bone tissue. Some of this may be due to the method of delivery. In the case of chondroitin sulfate and glucosamine, it is likely that not all of the dose was absorbed due to loss during digestion. While these components may still act on the gut microbiome and reduce inflammation, an intradermal or intraperitoneal injection would deliver a higher dose.

There were some subtle differences in structural bone properties, primarily observed in the femur. In the cortical bone, endosteal circumference and marrow area were higher in glucosamine-treated mice compared to controls. These differences did not result in differences to cortical thickness or area, likely as the overall size of the bones expanded (both total cross sectional area and periosteal perimeter trended up and reached significance for the main ANOVA, but not in posthoc testing). The structural differences were too subtle to alter the overall mechanical properties, especially since tissue-level properties trended downward. In the tibiae, the only alteration was that trabecular thickness was lower in the trabeculae of chondroitin sulfate-treated mice. This did not correspond to a change in trabecular bone volume of the tibiae.

Other studies have indicated that dietary supplements do not improve bone’s fracture resistance. Rabbits a fed a dietary supplement of fish oil had lower cortical area than control rabbits, but also had lower energy intake than controls^31^. A second control group of pair-fed rabbits was included, but cortical area was still lower in the fish oil fed group compared to the pair fed group and the standard controls. Rabbits fed fish oil also had lower stress than standard control rabbits, but the rabbits that were pair-fed also had a lower stress than the standard controls, indicating that the negative effect of fish oil was not independent of caloric intake. The max load and total energy absorbed were lower in fish oil-fed rabbits compared to both control groups. For growing, healthy animals, the differences in caloric intake and its effects on the bone may outweigh the benefits that could be gained through supplementation. In our study, while food intake was not measured, the final body weights did not differ in any group, making it less likely that there were significant differences in caloric intake.

In contrast, other studies have shown improvements to bone with dietary supplementation. Ovariectomized rats given a high dose of hydrolyzed collagen had increased maximum load in the vertebrae, but not the femur^32^. Unfortunately, there was no microCT analysis done in this study, but the mass of the vertebrae was higher than the other ovariectomized groups indicating possible increases in bone volume fraction. N-acetyl-D-glucosamine derived from chitin was used to treat ovariectomized Sprague Dawley rats. These rats had a higher ash fraction in their bone and higher maximum load^38^. Fish oil supplementation has been found to prevent a loss in BV/TV in the vertebrae with age in female C57BL/6 J mice^30^. It should be noted that in that study, all dietary fat was from fish oil, which constituted 22% of the diet composition. This is significantly higher than what would be used in a supplement. The improvement was also dependent on mouse genetic strain^30^. Overall, it appears that dietary supplements may improve bone quality only in cases where the bone quality has been previously decreased such as with estrogen loss or aging. These are also conditions in which inflammation increases and bound water may decrease, areas in which dietary supplements may be of more assistance.

Some limitations of this study should be noted. The method of delivery the supplements was used to reduce stress associated with gavage feeding. However, it also ensured that while the cage received an average dose as listed, there may have been variation in the dose each individual mouse received. The mice were observed to verify that all were eating the supplements in order to help control for this limitation. These animals were fed the supplements to mimic dietary supplementation, but adding more components to growing bone may work better by injection due to loss in the gut. The dosage in this study was determined by using the BSA formula. This technique does have its drawbacks as it does not take into differences in murine metabolism as discussed more in depth elsewhere^39^, but the lack of pharmacokinetic data for dietary supplements in mice prevented a more complex conversion. Human dosages were chosen based on typical amounts indicated for dietary supplements. These were chosen to be towards the high range in recommended amounts seen on various labels. The goal of this project was to determine whether dietary supplements would have any impact on bone quality, and dosages were chosen to be on the higher side to eliminate a possibility that low-dosage would affect the results. It should also be considered that mice were on a standard rodent chow, which is specifically designed to ensure that mice receive all their required nutrients. Humans do not always have balanced diets, and thus, supplements may be more helpful to individuals who are not achieving the recommended nutrients through diet. Lastly, mice were fed standard rodent chow ad libitum, and supplemented groups were not restricted in their diet to control for differences in caloric intake.

## Conclusion

Dietary supplements did not improve bone quality in growing, healthy female mice. There were subtle structural differences in the bone, which could be detrimental. These did not result in changes in the structural mechanical properties of bone, and in this study, the only material-level change to the mechanical properties of bone was a lower elastic modulus in glucosamine and fish oil-treated mice. Dietary supplements may be more beneficial in individuals without a balanced diet or in those with an increased risk of fracture, such as those experiencing estrogen loss.

## Supplementary Information

Supplementary Information 1.

## Data Availability

The datasets generated during and/or analyzed during the current study are available from the corresponding author on reasonable request.

## References

[1] Rho J-Y, Kuhn-Spearing L, Zioupos P (1998). Mechanical properties and the hierarchical structure of bone. Med. Eng. Phys..

[2] Morgan S, Poundarik AA, Vashishth D (2015). Do non-collagenous proteins affect skeletal mechanical properties?. Calcif. Tissue Int..

[3] Shoulders MD, Raines RT (2009). Collagen structure and stability. Ann. Rev. Biochem..

[4] Granke M, Does MD, Nyman JS (2015). The role of water compartments in the material properties of cortical bone. Calcif. Tissue Int..

[5] Hua R (2020). Biglycan and chondroitin sulfate play pivotal roles in bone toughness via retaining bound water in bone mineral matrix. Matrix Biol..

[6] Currey J (1984). Effects of differences in mineralization on the mechanical properties of bone. Philos. Trans. R. Soc. B.

[7] Zioupos P, Currey JD, Casinos Adria (2000). Exploring the effects of hyperminerlisation in bone tissue by using an extreme biological example. Connect. Tissue Res..

[8] Burton B (2014). Bone embrittlement and collagen modifications due to high-dose gamma-irradiation sterilization. Bone.

[9] Odetti P, Rossi S, Monacelli F, Poggi A, Cirnigliaro M, Federici M, Federici A (2005). Advanced glycation end products and bone loss during aging. Ann. N. Y. Acad. Sci..

[10] Ruiz HH, Ramasamy R, Schmidt AM (2020). Advanced glycation end products: building on the concept of the "common soil" in metabolic disease. Endocrinology.

[11] Yamamoto M, Sugimoto T (2016). Advanced glycation end products, diabetes, and bone strength. Curr Osteoporos Rep.

[12] Schulman RC, Weiss AJ, Mechanick JI (2011). Nutrition, bone, and aging: An integrative physiology approach. Curr Osteoporos Rep.

[13] Palacios C (2006). The role of nutrients in bone health, from A to Z. Crit. Rev. Food Sci. Nutr..

[14] Miller KL, Clegg DO (2011). Glucosamine and chondroitin sulfate. Rheum. Dis. Clin..

[15] Simental-Mendía M (2018). Effect of glucosamine and chondroitin sulfate in symptomatic knee osteoarthritis: A systematic review and meta-analysis of randomized placebo-controlled trials. Rheumatol. Int..

[16] Henrotin Y, Marty M, Mobasheri A (2014). What is the current status of chondroitin sulfate and glucosamine for the treatment of knee osteoarthritis?. Maturitas.

[17] Stabler TV, Huang Z, Montell E, Vergés J, Kraus VB (2017). Chondroitin sulphate inhibits NF-κB activity induced by interaction of pathogenic and damage associated molecules. Osteoarthr. Cartil..

[18] Gouze JN (2002). Glucosamine modulates IL-1-induced activation of rat chondrocytes at a receptor level, and by inhibiting the NF-κB pathway. FEBS Lett..

[19] Boyce BF, Yao Z, Xing L (2010). Functions of nuclear factor kappaB in bone. Ann N Y Acad Sci.

[20] Volpi N (2019). Oral bioavailability and pharmacokinetics of nonanimal chondroitin sulfate and its constituents in healthy male volunteers. Clin. Pharmacol. Drug Dev..

[21] Ibrahim A, Gilzad-kohan MH, Aghazadeh-Habashi A, Jamali F (2012). Absorption and bioavailability of glucosamine in the rat. J. Pharm. Sci..

[22] Moon JM (2021). Impact of glucosamine supplementation on gut health. Nutrients.

[23] Comblain F, Serisier S, Barthelemy N, Balligand M, Henrotin Y (2016). Review of dietary supplements for the management of osteoarthritis in dogs in studies from 2004 to 2014. J. Vet. Pharmacol. Ther..

[24] König D, Oesser S, Scharla S, Zdzieblik D, Gollhofer A (2018). Specific collagen peptides improve bone mineral density and bone markers in postmenopausal women-a randomized controlled study. Nutrients.

[25] Clifford T (2019). The effects of collagen peptides on muscle damage, inflammation and bone turnover following exercise: A randomized, controlled trial. Amino Acids.

[26] Harkness ML, Harkness RD, Venn MF (1978). Digestion of native collagen in the gut. Gut.

[27] Saini RK, Keum Y-S (2018). Omega-3 and omega-6 polyunsaturated fatty acids: Dietary sources, metabolism, and significance — A review. Life Sci..

[28] Griel AE (2007). An increase in dietary n-3 fatty acids decreases a marker of bone resorption in humans. Nutr. J..

[29] Kim MG, Yang I, Lee HS, Lee JY, Kim K (2020). Lipid-modifying effects of krill oil vs fish oil: A network meta-analysis. Nutr Rev.

[30] Bonnet N, Somm E, Rosen CJ (2014). Diet and gene interactions influence the skeletal response to polyunsaturated fatty acids. Bone.

[31] Judex S (2000). Dietary fish oil supplementation adversely affects cortical bone morphology and biomechanics in growing rabbits. Calcif. Tissue Int..

[32] de Almeida Jackix E, Cúneo F, Amaya-Farfan J, de Assunção JV, Quintaes KD (2010). A food supplement of hydrolyzed collagen improves compositional and biodynamic characteristics of vertebrae in ovariectomized rats. J. Med. Food.

[33] Johnell OKJ (2005). Epidemiology of osteoporotic fractures. Osteoporos. Int..

[34] Nair AB, Jacob S (2016). A simple practice guide for dose conversion between animals and human. J. Basic Clin. Pharm..

[35] Turner CH, Burr DB (1993). Basic biomechanical measurements of bone: A tutorial. Bone.

[36] Ritchie R, Koester KJ, Ionova S, Yao W, Lane NE, Ager JW (2008). Measurement of the toughness of bone: A tutorial with special reference to small animals. Bone.

[37] Currey JD, Zioupos P, Davies P, Casinos A (2017). Mechanical properties of nacre and highly mineralized bone. Proc. R. Soc London B.

[38] Jiang Z (2018). Dietary natural N-Acetyl-d-glucosamine prevents bone loss in ovariectomized rat model of postmenopausal osteoporosis. Molecules.

[39] Blanchard OL, Smoliga JM (2015). Translating dosages from animal models to human clinical trials—revisiting body surface area scaling. FASEB J..

