# Supplementary figures and images for: Dietary supplements do not improve bone morphology or mechanical properties in young female C57BL/6 mice
Source: Sci Rep. 2022 Jun 13;12:9804. doi: 10.1038/s41598-022-14068-2 (PMC9192719; doi:10.1038/s41598-022-14068-2)

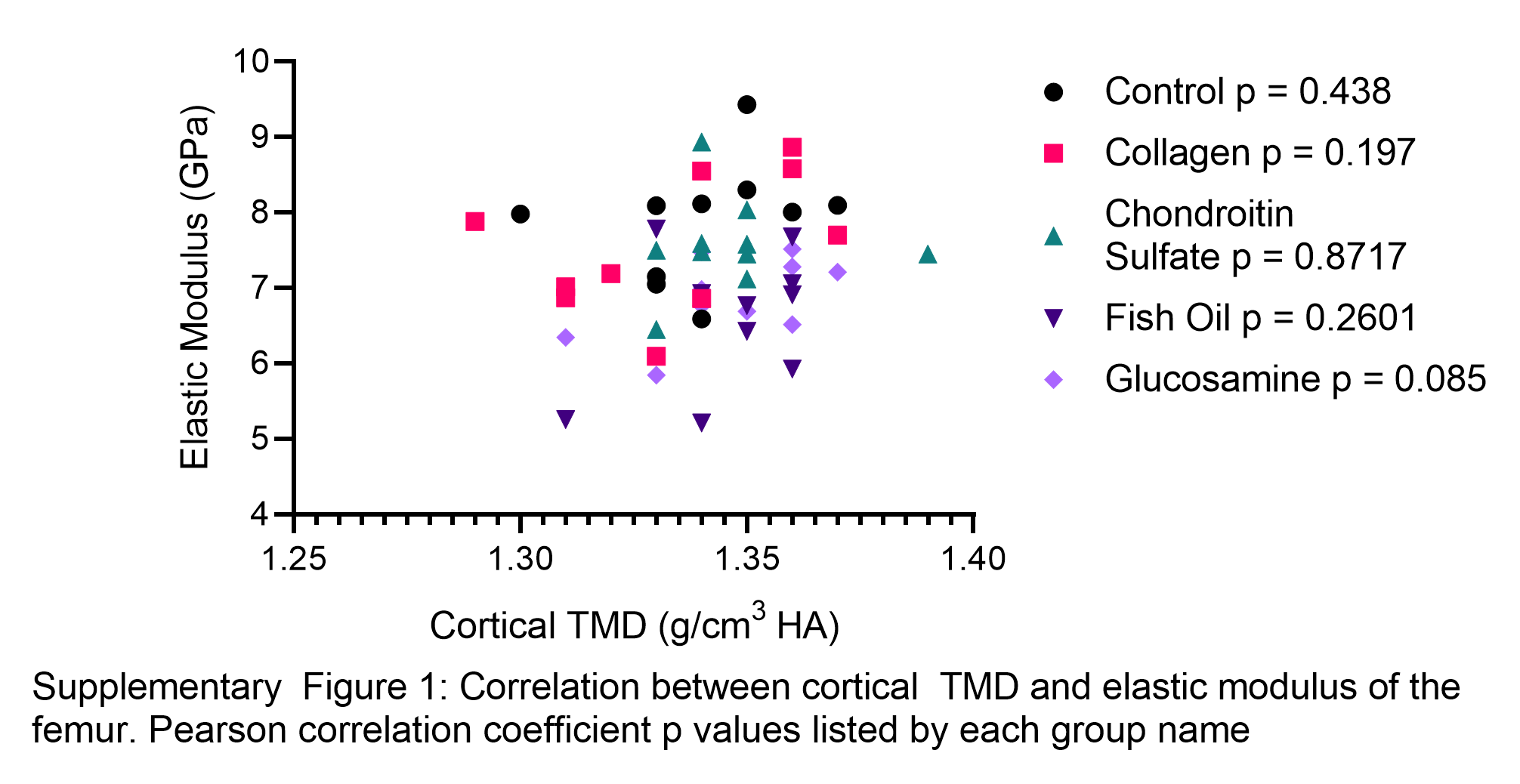

Supplement: Supplementary file 1 — Supplementary Information 1. [file 41598_2022_14068_MOESM1_ESM.tif]
